# Cetuximab-induced aseptic meningitis: case report and review of a rare adverse event

**DOI:** 10.1186/s12885-016-2434-7

**Published:** 2016-07-04

**Authors:** Christophe Maritaz, Carole Metz, Nabil Baba-Hamed, Méryam Jardin-Szucs, Gaël Deplanque

**Affiliations:** Department of Pharmacy, Saint-Joseph Hospital, Paris, France; Department of Oncology, Saint-Joseph Hospital, Paris, France; Department of Oncology, University hospital of Lausanne, Lausanne, Switzerland

**Keywords:** Cetuximab, Aseptic meningitis, Cancer

## Abstract

**Background:**

Cetuximab is a commonly used antibody agent in the treatment of colorectal or head and neck cancer. Although it is generally well tolerated in most patients, cetuximab has been associated with some rare but serious adverse events. Aseptic meningitis is one such distinctly uncommon adverse drug reaction.

**Case presentation:**

We present the case of a middle-aged Caucasian patient, who presented with fever and headache within a few hours of starting cetuximab therapy and was diagnosed with cetuximab-induced aseptic meningitis after a complete workup.

**Conclusion:**

To our knowledge, this is the ninth case of cetuximab-induced aseptic meningitis reported in literature. Because of a nonspecific clinical presentation, this adverse drug reaction can be easily misdiagnosed. It is important to increase awareness of this potentially severe reaction among oncologists.

## Background

Cetuximab, a human/mouse chimeric monoclonal antibody against the epidermal growth factor receptor (EGFR), is used as a single agent and in combination with chemotherapy or radiation therapy in metastatic colorectal cancer and locally advanced or metastatic head and neck squamous cell cancer. In cetuximab Summary of Product Characteristics (SPC), aseptic meningitis is mentioned as a rare nervous system disorder but with an unknown frequency. Rare but serious cancer drug-associated adverse reactions can be identified in the postmarketing experience after large numbers of patients have been exposed to the drug. As a rare complication, we report a case of aseptic meningitis associated with the first intravenous (I.V.) administration of cetuximab.

## Case presentation

A 66-year-old woman, with a WHO performance status of 0, history of chronic smoking, high blood pressure and atrial fibrillation, was diagnosed with a stage IVa locally advanced laryngeal squamous-cell carcinoma (cT3N2M0). She had neither history of headache nor previous allergic drug reactions. She received neoadjuvant chemotherapy by docetaxel, cisplatin and fluorouracil, with a marked tumor regression following three courses. She was then offered definitive external beam radiotherapy with concurrent weekly cetuximab. On her first cycle, she received routine premedication with dexchlorpheniramine 5 mg I.V. followed by a loading dose of 400 mg/m^2^ cetuximab I.V. over 2 h (5 mg/min) without developing any infusion reaction. Her usual medicines were rilmenidine, pantoprazole, fenofibrate, and acetaminophen. However, 4 h after completing cetuximab infusion, she was admitted to hospital with sudden headaches, photophobia, neck stiffness and vomiting without fever.

Cerebrospinal fluid (CSF) analysis showed a cloudy liquid with elevated protein (1.5 g/L; normal range: 0.2–0.4 g/L), a red blood cell count of 6/μL, and a leukocyte count of 4100/μL (normal range: 0–4/μL), 90 % of them were neutrophils, 9 % were lymphocytes, and 1 % were monocytes. The glucose level in CSF was 3.16 mM (normal range: 2.7–4.2 mM) with a glucose level in blood of 7.3 mM (ratio 0.43). The white blood cell count was 7900/μL with 7000/μL neutrophils, and a C-reactive protein at 5.9 mg/L (normal range <6.0 mg/L). The patient was treated with empiric antibiotic therapy (ceftriaxone I.V.) for 7 days without corticosteroids and recovered neurologically within 8 days. Bacterial cultures remained negative. Viral analysis including a viral encephalitis panel was performed by polymerase chain reaction and remained negative. Repeat CSF analysis was initially planned 8 days after admission to the hospital but the lumbar puncture failed and was not repeated as the patient was well.

Symptoms resolution was reported by day 2. Radiation therapy was started 3 weeks after for 8 weeks and cetuximab was reintroduced 28 days after with a lower dose of 250 mg/m^2^. Methylprednisolone 80 mg I.V. was added to dexchlorpheniramine 5 mg I.V. and the infusion flow rate of cetuximab was decreased to 2 mg/min. She tolerated it well and no side effects were reported all along the other additional infusions up to 10 weeks. At a follow-up of 18 months the patient is well with no evidence of tumor recurrence.

### Discussion

The temporal association, clinical and laboratory findings strongly support the diagnosis of cetuximab-induced aseptic meningitis. As for our patient, most patients with aseptic meningitis are treated with antibiotics, pending identification of infectious agent and recover within 2 weeks, without any long-term neurological sequelae.

Distinction on clinical grounds alone is not possible, and the CSF pattern with neutrophilic pleocytosis may cause confusion with infectious meningitis. Resolution occurs several days after drug discontinuation. Diagnosis of aseptic meningitis is based on viral and bacterial CSF profiles remaining sterile.

Nonsteroidal anti-inflammatory drugs, antibiotics, intravenous immunoglobulins, antiepileptic drugs, and monoclonal antibodies (mainly tumor necrosis factor inhibitors) are the most frequent cause of drug-induced meningitis. History of drug intake is crucial because there are no specific characteristics associated with a specific drug [1].

In order to try to understand the pathophysiology of aseptic meningitis due to cetuximab, we can draw similarities with aseptic meningitis occurring with I.V. immunoglobulin (IVIG) infusion [2–6]. The factors, which may predispose to the development of the meningitis, include fast infusion rates and a history of headaches. The symptoms of aseptic meningitis generally occur within 24 h of starting treatment. Theories of aseptic meningitis with IVIG have included an allergic hypersensitivity reaction or serum immunoglobulin crossing the blood brain barrier. Hence, this entry of serum immunoglobulin into the cerebrospinal fluid would be responsible for the inflammatory reaction. It has also been suggested that releasing histamine, serotonin, and prostaglandins could affect the meningeal microvasculature, such as in migraine mechanism [7].

The first occurrence of drug-induced aseptic meningitis related to cetuximab was reported in 2000 by Baselga et al. in a phase I clinical trial [8]. Since then, 7 other cases of cetuximab-induced aseptic meningitis have been described. Cetuximab was reintroduced successfully for three of them with an appropriate premedication and a slower infusion rate, one patient had a positive rechallenge without corticosteroid premedication [9, 10]. Characteristics of the reported patients from the literature were compiled recently [11] and are now completed with a new one [12] and our present report in Table 1. Note that these adverse reactions always occurred during the first administration which may suggest a dose-related response, even though an idiosyncratic response in patients with risk factors is also possible. Surprisingly, there are no cases described in colorectal cancer whereas cetuximab is commonly being dosed at 500 mg/m^2^ (higher dose) every 2 weeks for a larger number of patients.

**Table 1:** Characteristics of the described cases of cetuximab-induced aseptic meningitis

Case, Date	Age range	Indication for cetuximab	Cetuximab dose (duration), premedication	Symptoms (time onset), imaging	Initial CSF analysis	Follow-up CSF analysis	Treatment, recovery	Rechallenge
1, 2000 [8]	N/R	N/R	100 mg/m^2^	N/R	N/A	N/A	N/R	N/R
2, 2009 [10]	40–49	Recurrent laryngeal squamous cell carcinoma	400 mg/m^2^ (first administration 2 h), diphenhydramine 50 mg IV	Frontal headache, 38.9 °C fever (few hours after infusion), N/R	2300/μl with 98 % neutrophils, protein 1.04 g/L, normal glucose level, negative cultures	“Resolution of neutrophilic pleocytosis”, normal protein levels (day 4)	Empirical antibiotic treatment, acyclovir, recovery N/R	Negative rechallenge after 1 week (250 mg/m^2^, premedication: dexamethasone, diphenhydramine) without adverse events
3, 2009 [10]	40–49	Locally advanced squamous cell carcinoma of right tonsil	400 mg/m^2^ (first administration 2 h), diphenhydramine 50 mg IV	Severe frontal headache, 39.4 °C fever, neck stiffness, photophobia (about 8 h after infusion), N/R	2267/μl with 90 % neutrophils, protein 1.46 g/L, normal glucose level, negative cultures	“No white blood cells”, elevated but improved protein (0.69 g/L)	Empirical antibiotic treatment, acyclovir, dexamethasone, recovery from meningeal symptoms after 12 days	Negative rechallenge after 2 weeks (250 mg/m^2^, premedication: dexamethasone, diphenhydramine, famotidine) without adverse events
4, 2010 [13]	70–79	NSCLC (stage IIIA)	400 mg/m^2^ (first administration, duration N/R), N/R	Severe headache, nausea, vomiting, neck stiffness (few hours after infusion), brain CT scan normal	528/μl with 87 % neutrophils, “modestly elevated protein”, normal glucose level	N/A	Empirical antibiotic treatment (stopped after infection was ruled out), recovery without neurological sequelae	N/R
5, 2010 [13]	50–59	Metastatic NSCLC	400 mg/m^2^ (first administration, duration N/R), N/R	Acute encephalopathy (few hours after infusion), brain CT scan and MRI normal	cell count and fraction of neutrophils N/A, protein 1.16 g/L, glucose 2.8 mmol/L, negative cultures	N/A	Empirical antibiotic treatment (stopped after infection was ruled out), recovery within several days	N/R
6, 2012 [9]	50–59	Squamous maxillary cancer (stage IVb)	400 mg/m^2^ (first administration), diphenhydramine 50 mg IV	Frontal headache, neck discomfort, 39.9 °C fever (few hours after infusion), brain CT scan normal	1025/μl with 92 % neutrophils, protein 1.65 g/L, normal glucose level, negative bacterial culture, PCR (HSV) negative	N/A	Empirical antibiotic treatment, resolution of symptoms – no complications.	Positive rechallenge after 4 weeks (250 mg/m^2^), recurrent CSF pleiocytosis (715/μl, 93 % neutrophils), protein 1.22 g/L, premedication: diphenhydramine. Rechallenge three and following without adverse events.
7, 2015 [11]	60–69	Recurrent advanced oropharyngeal squamous cell carcinoma	400 mg/m^2^ (first administration, 2 h), clemastine 2 mg oral	Headache, mutism, hypertension, neck stiffness, 39.2 °C fever (about 9 h after infusion), brain CT scan and MRI non-diagnostic	1413/μl with 92 % neutrophils, protein 1.79 g/L, normal glucose level 3.5 mmol/L, negative cultures and serologies	Cell count 1/μl, protein 0.68 g/L, normal glucose level 4.0 mmol/L	Empirical antibiotic treatment, dexamethasone (stopped after infection was ruled out), myoclonic jerks and NCSE after 3 days, recovery within 14 days	The patient refused rechallenge.
8, 2015 [12]	50–59	Tonsillar squamous cell cancer	400 mg/m^2^ (first administration)	Frontal headache (10/10 in severity), fever (1 h after infusion), brain CT scan	473/μl with 80 % neutrophils in tube 1 and 500/μl with 62 % neutrophilsin tube 4. 150 and 50 cells/μL red blood cells, protein 1,28 g/L, normal glucose level	N/A	Empirical antibiotic treatment for 4 days (stopped after infection was ruled out). Symptomatic improvement after 2 days and recovery within 4 days	Negative rechallenge after 7 days, the patient received a second dose of cetuximab at 250 mg/m2 without adverse events.
9, 2015 Present case	60–69	Locally advanced laryngeal squamous cell carcinoma	400 mg/m^2^ (first administration, 2 h), dexchlorpheniramine 5 mg IV	Headache, photophobia, neck stiffness, vomiting, nausea (few hours after infusion), N/A	Leukocytes count 4100/μL with 90 % of neutrophils, 6/μL red blood cells, protein 1.5 g/L, normal glucose level in 3.16 mmol/L., negative viral and bacterial cultures.	N/A	Empirical antibiotic treatment for 7 days, recovery without sequelae within several days	Negative rechallenge after 28 days (250 mg/m^2^, premedication: methylprednisolone, dexchlorpheniramine) without adverse events.

## Conclusions

Cetuximab-induced aseptic meningitis should be known as a potential severe adverse drug reaction with corticosteroids introduced before loading dose and slowed infusion. This has been taken into account in the 2014 SPC renewal, as « Prior to the first infusion, patients must receive premedication with an antihistamine and a corticosteroid at least 1 h prior to administration of cetuximab. This premedication is recommended prior to all subsequent infusions » and « The initial dose should be given slowly and speed of infusion must not exceed 5 mg/min (1 ml/min). For the subsequent doses, the infusion rate must not exceed 10 mg/min (2 ml/min) ». This report added to the others may serve as a reference for health practitioners managing cetuximab. Rechallenge with cetuximab after complete neurological resolution is feasible and should be attempted, especially when cetuximab is given in a curative intent.

## Abbreviations

EGFR, epidermal growth factor receptor; SPC, summary of product characteristics; IV, intravenous; WHO, World Health Organization; CSF, cerebrospinal fluid
